# Acute Appendicitis at Stem Cell Infusion During Autologous Transplantation for HIV-Associated Diffuse Large B-cell Lymphoma: A Case Report

**DOI:** 10.7759/cureus.110505

**Published:** 2026-06-09

**Authors:** Mossaab El Mousadik, Adnane Bousokri, Tahar Tazi, Monsif Fadi, Halima Hadri

**Affiliations:** 1 Clinical Hematology and Cellular Therapy, Centre Hospitalier Universitaire Mohammed VI d'Agadir, Agadir, MAR; 2 Hematology, Hôpital Universitaire International Cheikh Khalifa Ibn Zaid, Casablanca, MAR; 3 Visceral Surgery, Hôpital Universitaire International Cheikh Khalifa Ibn Zaid, Casablanca, MAR; 4 Pathology, Hôpital Universitaire International Cheikh Khalifa Ibn Zaid, Casablanca, MAR; 5 Oncopathology, Cancer Biology and Environment Laboratory, Mohammed VI University of Health and Sciences (UM6SS), Casablanca, MAR; 6 Hematology, Mohammed VI University of Health and Sciences (UM6SS), Casablanca, MAR

**Keywords:** acute appendicitis, astc, autologous stem cell transplantation, beam, diffuse large b-cell lymphoma, hiv-associated lymphoma

## Abstract

Acute appendicitis is an exceptionally rare complication of autologous hematopoietic stem cell transplantation (HSCT) and represents a diagnostic challenge, since its presentation overlaps with conditioning-related gastrointestinal toxicity and neutropenic enterocolitis. Published data are limited to isolated case reports, and its occurrence during autologous stem cell transplantation (ASCT) for human immunodeficiency virus (HIV)-associated lymphoma has not previously been described.

We report the case of a 39-year-old man with HIV-associated diffuse large B-cell lymphoma (DLBCL), non-germinal center subtype, Ann Arbor stage IV with gastric involvement, and a high-risk age-adjusted International Prognostic Index (score three). After failure of first-line rituximab, cyclophosphamide, doxorubicin, vincristine, and prednisone (R-CHOP) with a Deauville score of four on interim positron emission tomography-computed tomography, complete metabolic remission was achieved with two cycles of second-line rituximab, dexamethasone, cytarabine, and carboplatin (R-DHAC). The patient then proceeded to carmustine, etoposide, cytarabine, and melphalan (BEAM)-conditioned ASCT under controlled HIV viral load and persistent CD4 lymphopenia. On the day of stem cell infusion (day zero), he developed right lower quadrant tenderness associated with elevated inflammatory markers; abdominopelvic computed tomography demonstrated acute appendicitis. Emergency laparoscopic appendectomy revealed ulcerative appendicitis without tumor infiltration on histopathology. The postoperative course during aplasia was complicated by septic shock due to multidrug-resistant *Pseudomonas aeruginosa* bacteremia, with a favorable evolution to broad-spectrum antibiotic therapy and vasopressor support. Neutrophil engraftment occurred on day +14, and the patient was discharged on day +20, with no further complications and a complete remission maintained at six months of follow-up.

This case illustrates that acute appendicitis may occur as early as the day of stem cell infusion. Its presentation may be initially indistinguishable from melphalan-induced gastrointestinal toxicity, which makes early abdominopelvic CT indispensable for an early diagnosis. It also shows that, even when surgical source control is timely, profound aplasia and the HIV-related CD4 lymphopenia predispose to severe infectious complications. This justifies anticipatory broad-spectrum coverage adapted to local multidrug-resistant epidemiology and close multidisciplinary monitoring throughout the aplastic phase.

## Introduction

Diffuse large B-cell lymphoma (DLBCL) is the most common subtype of non-Hodgkin lymphoma, accounting for 20-50% of cases worldwide, with an approximate incidence of 7.2 per 100,000 persons per year. Its occurrence is strongly associated with immunodeficiency, particularly human immunodeficiency virus (HIV) infection [1]. In the era of combination antiretroviral therapy, the prognosis of HIV-associated DLBCL has improved, with five-year overall survival rates around 60-70% reported with rituximab-based immunochemotherapy. Outcomes are influenced by immune status (CD4 count), HIV viral load, and tumor biology, particularly the cell-of-origin subtype [2]. Autologous stem cell transplantation (ASCT) is an established option for chemosensitive relapsed or refractory disease, with studies demonstrating feasibility and outcomes comparable to those observed in HIV-negative patients [3].

Gastrointestinal complications are common after transplantation, owing to mucosal injury and neutropenia, and often present with nonspecific abdominal symptoms [4]. Acute appendicitis during ASCT is exceptionally rare, with only isolated cases reported [5-9]. Although appendicitis appears more frequently in HIV-infected individuals than in the general population [10], its occurrence during ASCT for HIV-associated lymphoma has not, to our knowledge, been reported. We describe a case of acute appendicitis occurring during carmustine or BCNU, etoposide, cytarabine and melphalan (BEAM)-conditioned ASCT in a patient with HIV-associated DLBCL.

## Case presentation

We report the case of a 39-year-old man diagnosed with DLBCL, non-germinal center subtype, concomitant with HIV infection. The disease was stage IV according to the Ann Arbor classification [11], with gastric involvement, and the age-adjusted International Prognostic Index indicated high-risk disease (score of three) [12]. Antiretroviral (ARV) therapy with dolutegravir, lamivudine, and tenofovir was initiated.

First-line treatment consisted of four cycles of rituximab, cyclophosphamide, doxorubicin, vincristine, and prednisone (R-CHOP) every 21 days. Interim positron emission tomography-computed tomography (PET-CT) showed residual metabolic activity with a Deauville score of four [13]. Second-line treatment with rituximab, dexamethasone, cytarabine, and carboplatin (R-DHAC) achieved complete metabolic remission after two cycles (Deauville score three), and the patient was considered eligible for ASCT.

At admission before transplantation, laboratory investigations showed lymphopenia with a low CD4 count and a controlled HIV viral load (<40 copies/mL). Liver tests demonstrated a cholestatic pattern (Table 1).

**Table 1:** Baseline laboratory findings prior to autologous transplantation The reference ranges in this table are based on commonly accepted adult clinical laboratory standards and may vary depending on institutional and regional laboratory practices. AST, Aspartate aminotransferase; ALT, Alanine aminotransferase; GGT, Gamma-glutamyl transferase; ALP, Alkaline phosphatase; HBsAg, Hepatitis B surface antigen; Anti-HBc, Hepatitis B core antibody; CD4, Cluster of differentiation 4 T lymphocytes; CD8, Cluster of differentiation 8 T lymphocytes; HIV, Human immunodeficiency virus.

Parameter	Result	Reference range
Hemoglobin (g/dL)	13.8	13.0-17.0
Platelets (×10^9^/L)	180	150-400
White blood cells (×10^9^/L)	2.79	4.0-10.0
Neutrophils (×10^9^/L)	1.57	1.5-7.5
Lymphocytes (×10^9^/L)	0.61	1.0-4.0
Total bilirubin (mg/L)	12	3-12
AST (IU/L)	56	10-40
ALT (IU/L)	62	7-56
GGT (IU/L)	286	9-48
ALP (IU/L)	241	44-147
Hepatitis B serology	HBsAg negative / anti-HBc positive	-
Hepatitis C serology	Negative	-
Toxoplasmosis serology	Negative	-
Syphilis serology	Negative	-
Cytomegalovirus viral load	Undetectable	Undetectable
CD4 (cells/mm^3^)	127	507-955
CD8 (cells/mm^3^)	379	404-826
HIV viral load (copies/mL)	Detected (not quantified), <40	Undetectable

Drug-induced liver injury was considered the most likely diagnosis after the exclusion of infectious causes, and ursodeoxycholic acid was initiated on day -8 of conditioning. Antiviral and anti-*Pneumocystis jirovecii* prophylaxis with valacyclovir, triple combination antiretroviral (ARV) therapy, and trimethoprim-sulfamethoxazole were maintained throughout transplantation.

The stem cell mobilization was done with filgrastim and plerixafor, yielding 3.7 × 10^6^ CD34+ cells/kg. Conditioning consisted of the BEAM regimen: carmustine 300 mg/m^2^ on day -6, etoposide 100 mg/m^2^ every 12 hours from day -5 to day -2, cytarabine 100 mg/m^2^ every 12 hours from day -5 to day -2, and melphalan 140 mg/m^2^ on day -1.

**Figure 1:**
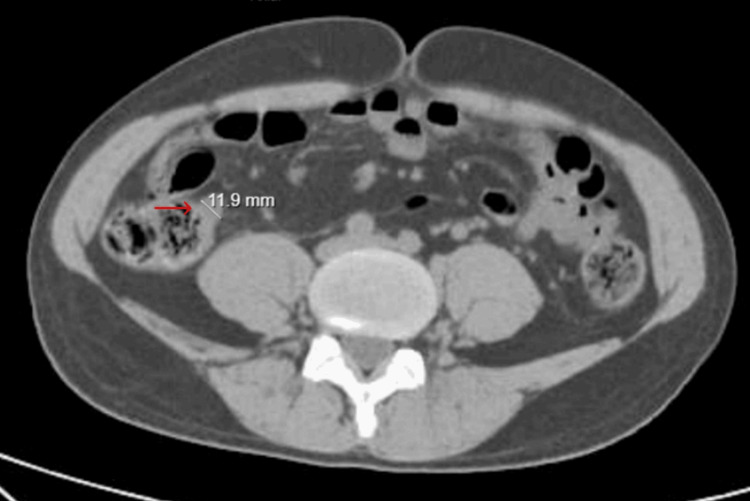
Abdominopelvic computed tomography (CT) findings Axial contrast-enhanced abdominal CT scan showing an enlarged appendix measuring approximately 11.9 mm in diameter with peri-appendiceal fat stranding (arrow), consistent with acute appendicitis diagnosis.

On day -1, the patient developed a mild and well tolerated abdominal pain associated with constipation and without fever, which was at first attributed to melphalan-related gastrointestinal toxicity. Stem cell reinfusion was performed on day zero. Later that same day, right lower quadrant tenderness appeared, with a concomitant rise in inflammatory markers. Abdominopelvic computed tomography revealed an enlarged appendix measuring approximately 11.9 mm with peri-appendiceal fat stranding, consistent with acute appendicitis (Figure 1).

**Figure 2:**
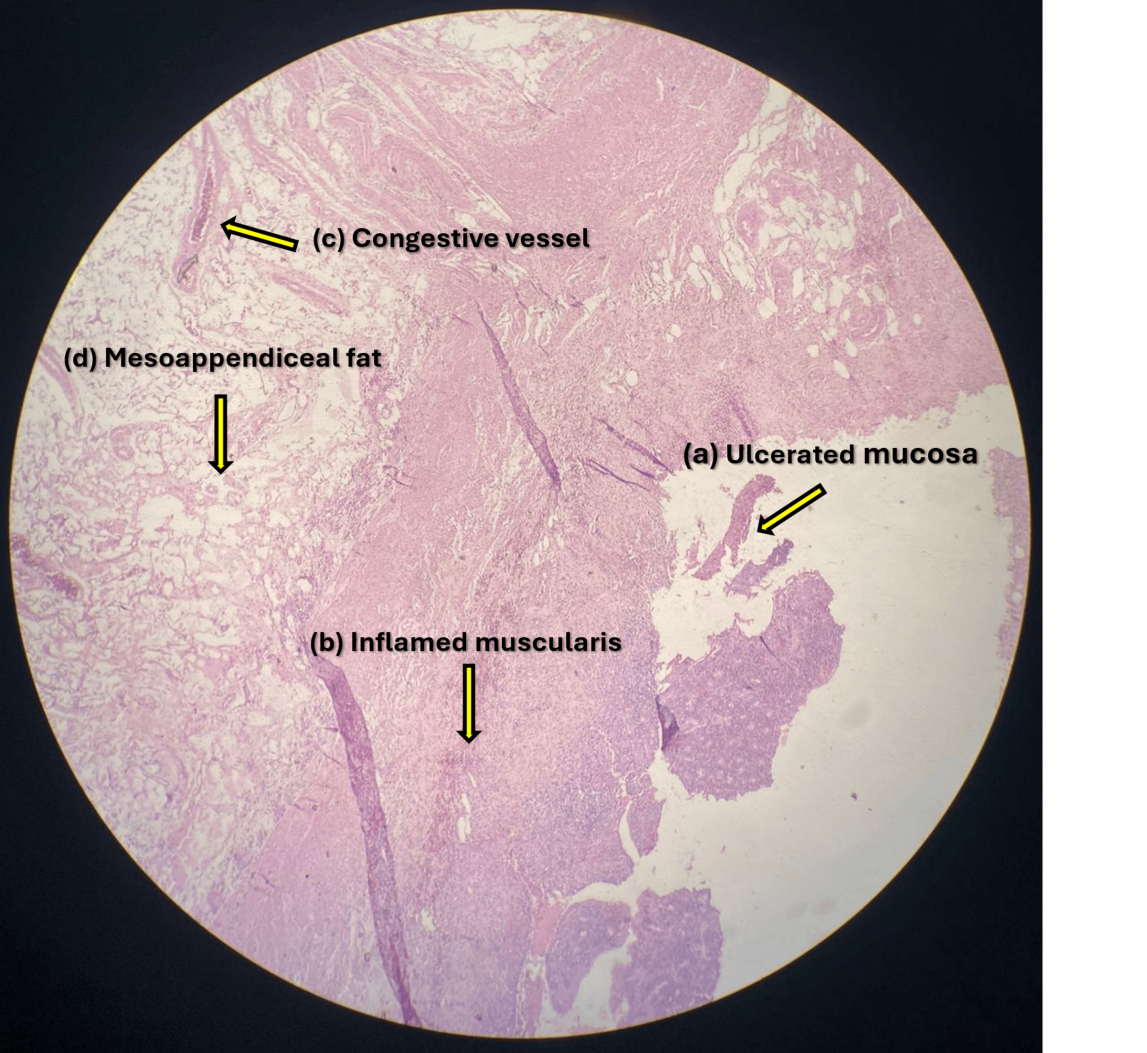
Histopathological appearance of the appendix Image after hematoxylin and eosin staining (original magnification ×4). The mucosal lining shows extensive ulceration with loss of the normal glandular architecture (a). The acute inflammatory infiltrate extends transmurally through an edematous and congested muscularis propria (b), with scattered inflammatory cells visible within the smooth muscle layer. A congested vessel within the appendiceal wall is identified (c), consistent with the marked vascular congestion typically observed in acute appendicitis. Inflammation reaches the mesoappendiceal adipose tissue (d), where adipocytes appear separated by edema and infiltrated by inflammatory cells. These findings are consistent with acute ulcerative appendicitis with periappendicitis.

The patient underwent laparoscopic appendectomy. Intraoperatively, the appendix was markedly inflamed with a friable base, which required stapled closure at the caecal base. Peritoneal lavage was performed, and a drain was placed. Histopathological examination showed ulcerative appendicitis and no tumor infiltration (Figure 2).

Postoperatively, ceftazidime plus metronidazole was initiated. On day five, neutropenic fever occurred during agranulocytosis, which prompted escalation to meropenem and vancomycin with continuation of metronidazole; fluconazole prophylaxis and filgrastim support were also started. Colistin was added on day seven because of persistent fever. On day nine, a perianal phlegmon was identified. The following day, the patient developed septic shock complicated by disseminated intravascular coagulation. Peripheral blood cultures isolated multidrug-resistant *Pseudomonas aeruginosa*, susceptible only to colistin. Antimicrobial therapy was further intensified with tigecycline and ceftazidime-avibactam while colistin was maintained, and fluconazole was replaced by voriconazole. Norepinephrine support was required for 48 hours. Clinical improvement and apyrexia were observed from day +12, with progressive reduction of inflammatory markers and neutrophil recovery on day +14 (Table 2).

**Table 2:** Evolution of inflammatory markers and clinical events during autologous stem cell transplantation (ASCT) ^*^Day relative to stem cell infusion (day 0); ^**^Platelet concentrate transfusions administered; ^†^Normal CRP: <5 mg/L; ^‡^Normal procalcitonin: <0.05 ng/mL; ^§^Normal neutrophil count: 1,500–7,500/mm³; ^¶^Normal platelet count: 150,000–450,000/mm³. The reference ranges in this table are based on commonly accepted adult clinical laboratory standards and may vary depending on institutional and regional laboratory practices.

Day^*^	CRP^†^ (mg/L)	Procalcitonin^‡^ (ng/mL)	Neutrophils^§^ (/mm^3^)	Platelets^¶^ (/mm^3^)	Infectious event
0	21	-	2,570	87,000	Acute appendicitis
+1	60	-	100	89,000	-
+3	101	-	0	32,000	-
+5	113	0.19	0	17,000	Neutropenic fever
+7	167	-	0	8,000^**^	-
+9	162	0.8	0	5,000	Perianal phlegmon
+10	225	0.65	0	2,000^**^	Septic shock
+11	204	7.67	-	-	-
+12	107	2.54	420	18,000	Apyrexia
+14	-	-	1,360	4,000^**^	Neutrophil recovery
+15	53.7	0.82	2,750	17,000	-
+20	6.50	-	5,600	28,000	Discharge

Antimicrobial therapy was subsequently de-escalated, and the patient was discharged on day +20. At six months of follow-up, he remained alive in complete remission without evidence of relapse.

## Discussion

Acute appendicitis has traditionally been attributed to luminal obstruction, causing increased intraluminal pressure, impaired perfusion, bacterial overgrowth, and progressive inflammation that may or may not lead to gangrene or perforation. Contemporary evidence suggests that microbial dysbiosis and host immune responses also contribute to disease severity [14]. Immunocompromised states such as post-chemotherapy aplasia, hematopoietic stem cell transplantation (HSCT) conditioning, and HIV infection may therefore facilitate appendiceal inflammation through mucosal barrier injury, neutropenia, microbiota disruption, and immune dysregulation, while also altering its clinical presentation.

In the setting of HSCT, the evaluation of abdominal pain is particularly challenging because conditioning regimens and post-transplant aplasia may alter the usual clinical and inflammatory presentation. In ASCT, high-dose melphalan conditioning commonly induces gastrointestinal mucosal toxicity, including nausea, vomiting, diarrhea, and abdominal pain, which may mimic an acute surgical abdomen or early appendicitis [15]. Other causes of abdominal pain vary depending on the pain pattern in patients undergoing auto- or allo-HSCT. Diffuse abdominal pain may suggest treatment-related mucosal toxicity, infectious colitis such as *Clostridioides difficile* colitis, cytomegalovirus (CMV) gastrointestinal infection, or gastrointestinal graft-versus-host disease, which presents with common clinical symptoms including diarrhea, fever, nausea, bleeding, or diffuse bowel inflammation. Localized right lower quadrant pain, in contrast, should raise suspicion for acute appendicitis, neutropenic enterocolitis (typhlitis), or appendiceal/caecal lymphomatous infiltration [4,16]. In this context, abdominal computed tomography (CT) is essential to distinguish focal appendiceal inflammation from diffuse ileocecal disease [4], while histopathological examination is necessary to exclude malignant infiltration or opportunistic infection.

In the present case, the occurrence of acute appendicitis shortly after melphalan conditioning suggests a potential role of chemotherapy-induced gastrointestinal mucosal injury in promoting local inflammation and bacterial translocation. In addition, persistent CD4 lymphopenia related to HIV infection, despite controlled viral replication, may have further increased susceptibility to appendiceal infection and inflammation [10].

Acute appendicitis during HSCT is exceedingly rare, with the literature limited to isolated case reports and small retrospective series showing heterogeneous clinical presentations and management strategies [5-9]. Cases have been described after melphalan-conditioned ASCT, during neutropenic aplasia after allogeneic HSCT, and more rarely in association with opportunistic infections such as CMV [5,7,9]. To our knowledge, this is the first reported case occurring during BEAM-conditioned ASCT in a patient with HIV-associated DLBCL.

Appendectomy remains the standard treatment for acute appendicitis in the general population. According to the 2020 World Society of Emergency Surgery guidelines, conservative management with broad-spectrum antibiotics may be considered in selected cases of uncomplicated appendicitis [17]. Evidence supporting this approach in profoundly immunocompromised patients, and particularly in post-HSCT patients, remains limited and is largely restricted to small retrospective series and case reports [5-9] (Table 3).

**Table 3:** Reported appendicitis/appendiceal disease during hematopoietic stem cell transplantation. ALL, acute lymphoblastic leukemia; allo-HSCT, allogeneic hematopoietic stem cell transplantation; ASCT, autologous stem cell transplantation; BEAM, carmustine, etoposide, cytarabine, and melphalan; CML, chronic myeloid leukemia; CMV, cytomegalovirus; DLBCL, diffuse large B-cell lymphoma; Flu-Cy-ATG, fludarabine-cyclophosphamide-antithymocyte globulin; GVHD, graft-versus-host disease; NR, not reported.

Study	Disease / transplant	Timing	Microbiology / histopathology	Management	Outcome
Forghieri et al., 2008 [5]	Multiple myeloma / melphalan-ASCT	Day 0	Negative microbiology; gangrenous appendicitis; no tumor identified	Appendectomy + antibiotics	Recovery
Wright et al., 2022 [6]	DLBCL / BEAM-ASCT; myeloma-plasma cell leukemia / melphalan-ASCT	Day +8, +9	Clostridioides difficile-associated appendiceal inflammation in 1 case; nonspecific appendiceal inflammation in 1 case	Antibiotics	Recovery
Zhang et al., 2018 [7]	ALL (n=7), CML (n=3) / allo-HSCT	Day -1 to +7	No pathogen or tumor identified	Antibiotics in all 10 cases; 1 delayed appendectomy	9 recoveries; 1 death
Alsharidah et al., 2022 [8]	Severe aplastic anemia / Flu-Cy-ATG allo-HSCT	Day -1	NR	Appendectomy + antibiotics	Recovery
Kothari et al., 2017 [9]	ALL / allo-HSCT with alemtuzumab for acute GVHD	Day +40	CMV appendicitis	Appendectomy + anti-CMV therapy	Recovery
Present case, 2026	HIV-associated DLBCL / BEAM-ASCT	Day 0	Ulcerative appendicitis; no tumor identified	Laparoscopic appendectomy + antibiotics	Recovery

Management of acute appendicitis during HSCT is particularly challenging. Surgery during the aplastic phase carries an increased perioperative bleeding risk due to thrombocytopenia [18], while profound neutropenia predisposes to postoperative sepsis and multidrug-resistant infections [5,7]. Conversely, delaying appendectomy or the failure of antibiotic therapy may result in perforation, diffuse peritonitis, and septic shock [14,17].

The currently available transplant literature illustrates this therapeutic uncertainty. Zhang et al. reported successful conservative management in all 10 allogeneic HSCT patients during aplasia, with only one delayed recurrence requiring appendectomy [7]. Forghieri et al., in contrast, described immediate surgical intervention for gangrenous appendicitis occurring on day zero after melphalan-conditioned ASCT [5]. In our patient, CT showed localized appendiceal disease, which supported early appendectomy before perforation. Despite surgery and broad-spectrum antibiotics, the patient developed severe infectious complications during aplasia, including multidrug-resistant Pseudomonas aeruginosa septic shock. This highlights the need for anticipatory broad-spectrum coverage adapted to local multidrug-resistant epidemiology and close multidisciplinary monitoring.

This report is limited by its single-case nature and by the very limited published literature, which mainly consists of isolated case reports and small retrospective series. As a result, strong evidence-based management recommendations remain difficult to establish.

## Conclusions

To our knowledge, we report the first documented case of acute appendicitis arising during BEAM-conditioned ASCT for HIV-associated DLBCL, with symptom onset on the day of stem cell infusion. Several important points can be highlighted. First, acute appendicitis may occur as early as day zero of ASCT and should be considered in the differential diagnosis of localized right lower quadrant pain, even when gastrointestinal toxicity related to conditioning chemotherapy appears to be the most plausible cause. Second, early abdominopelvic CT imaging should be strongly considered, given that clinical and laboratory findings may be attenuated by aplasia and can overlap with manifestations of neutropenic enterocolitis or chemotherapy-induced mucositis. Third, surgical source control alone may not be sufficient in this setting: HIV-associated CD4 lymphopenia combined with post-transplant neutropenia substantially increases the risk of multidrug-resistant infections, which supports the prompt initiation of broad-spectrum empiric antimicrobial therapy tailored to local epidemiology and the patient's infectious history, along with close multidisciplinary surveillance throughout the aplastic period. Further reports are warranted to better establish optimal management strategies for this highly immunocompromised population.
